# Cell Secretome: Basic Insights and Therapeutic Opportunities for CNS Disorders

**DOI:** 10.3390/ph13020031

**Published:** 2020-02-20

**Authors:** Andreia G. Pinho, Jorge R. Cibrão, Nuno A. Silva, Susana Monteiro, António J. Salgado

**Affiliations:** 1 Life and Health Sciences Research Institute (ICVS), School of Medicine, University of Minho, 4710-057 Braga, Portugal; PG33779@alunos.uminho.pt (A.G.P.); pg28737@alunos.uminho.pt (J.R.C.); nunosilva@med.uminho.pt (N.A.S.); susanamonteiro@med.uminho.pt (S.M.); 2 ICVS/3B’s PT Government Associate Laboratory, 4710-057 Braga/Guimarães, Portugal

**Keywords:** central nervous system, ischemic stroke, mesenchymal stem cells, Parkinson’s disease, regeneration, spinal cord injury, secretome, traumatic brain injury

## Abstract

Transplantation of stem cells, in particular mesenchymal stem cells (MSCs), stands as a promising therapy for trauma, stroke or neurodegenerative conditions such as spinal cord or traumatic brain injuries (SCI or TBI), ischemic stroke (IS), or Parkinson’s disease (PD). Over the last few years, cell transplantation-based approaches have started to focus on the use of cell byproducts, with a strong emphasis on cell secretome. Having this in mind, the present review discusses the current state of the art of secretome-based therapy applications in different central nervous system (CNS) pathologies. For this purpose, the following topics are discussed: (1) What are the main cell secretome sources, composition, and associated collection techniques; (2) Possible differences of the therapeutic potential of the protein and vesicular fraction of the secretome; and (3) Impact of the cell secretome on CNS-related problems such as SCI, TBI, IS, and PD. With this, we aim to clarify some of the main questions that currently exist in the field of secretome-based therapies and consequently gain new knowledge that may help in the clinical application of secretome in CNS disorders.

## 1. Introduction

The prevalence of people suffering from central nervous system (CNS) pathologies, such as spinal cord injury (SCI), traumatic brain injury (TBI), ischemic stroke (IS), and Parkinson’s disease (PD), is currently high (SCI: fluctuates between 8 per million in Spain to 83 per million in Alaska every year; TBI: 69 million cases every year; IS: more than 2 million young adults (18–50 years) yearly; PD: varying from 88 in Asia to 228 in South America for people aged 50–59 years) [1,2,3,4]. 

Although having different aetiology, symptomatology, and pathophysiology, CNS injuries and disorders are in part challenging due to a shared feature—the low regenerative capacity of the adult CNS [5,6]. Self and spontaneous repair of the CNS can eventually occur, although at a small scale, through the action of endogenous neural stem cells [7]. These cells can be found in the adult CNS, however only in specific small niches and in limited numbers, resulting in a low regenerative ability for fully repairing damaged tissue [8]. 

In addition to the lower intrinsic regenerative capability of the CNS, insults to its delicate structure, caused by trauma or injury, infections, stroke, or degeneration, often trigger several biological pathways (e.g., cellular dysfunction or death, overactivation of immune cells, production of reactive oxygen species or proteoglycans, and glial scar formation) that exacerbate the primary insult [9,10]. The reduced regeneration of neural tissue together with the development of these secondary events aggravate the initial injury collectively, contributing to serious neurological sequels. 

Currently, the clinical approach for most of the CNS disorders essentially consists in palliative care combined with drugs (when available), which only stabilize the lesion progression. Effective treatments to stop, reverse the neurodegenerative processes, and promote functional regeneration are lacking, contributing to the high prevalence observed.

Candidate novel therapies have emerged in the CNS regeneration context, such as molecular therapies [11,12], biomaterials [13], and cellular transplantation [14,15,16]. The last one, cell-based therapies, have brought a particular interest to the CNS regeneration field and have been proposed over the last decades as a possible route to repair damaged circuitries within the CNS. Several cellular types have already been tested in a broad number of models of CNS disorders aiming to repopulate the damaged tissue or minimize the secondary impacts of each pathology [16]. Despite their promising results, there are still some challenges that need to be solved, namely transplantation safety, efficiency, and mechanisms of action. Currently, a growing body of evidence points to the importance of the paracrine signaling as a mechanism to support recovery by the transplanted cells. For instance, it has been demonstrated that the beneficial effects observed upon mesenchymal stem cell (MSC) grafts on the injured CNS were mainly due to secreted factors rather than cellular engraftment, in models of spinal cord injury [14] and Parkinson’s disease [17], as reviewed elsewhere [18,19,20]. This new paradigm regarding the paracrine action instead of cellular replacement or cell–cell interrelation has originated a new possible therapeutic opportunity, using the cellular secretome to tackle diverse pathologies. 

In this review, we discuss the latest advances in the secretome field and its application to CNS disorders.

## 2. Cell Secretome-Based Therapy

MSC-based therapies have shown promising results for the treatment of CNS-related diseases such as SCI [21,22,23], TBI [24,25], IS [26,27,28], and PD [20]. Although the mechanism of action remains to be completely understood. Different studies point to the secreted factors and vesicles—their secretome—as agents for the regenerative effect rather than cellular differentiation [14]. 

The term secretome was introduced by Tjalsma et al. [29], who defined it as “both the components of machineries for protein secretion and the native secreted proteins”. This definition then evolved to a more elaborated concept by Hathout and Agrawal in which the secretome was defined by the factors that are secreted by a cell, tissue, or organism to the extracellular space under a defined time and conditions [30,31]. Currently, this definition could be updated since it is known that, beyond soluble factors, the secretome also has the presence of lipids and extracellular vesicles (EVs) carrying important molecules [32,33]. 

**Figure 1:**
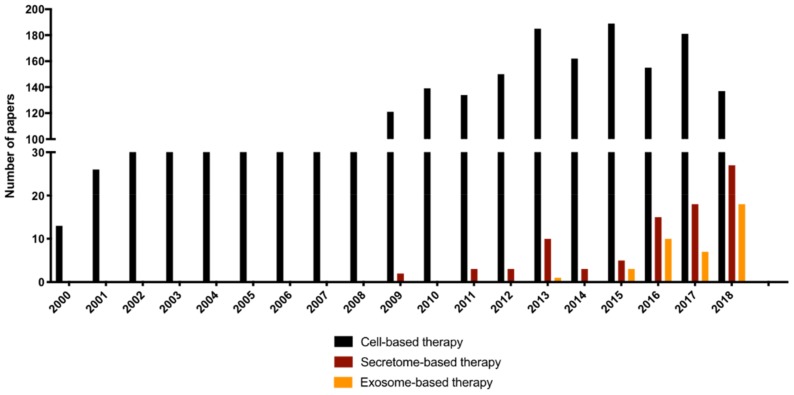
Number of publications per year of cell-, secretome-, or exosome-based therapies on central nervous system (CNS) disorders (Parkinson’s disease (PD), spinal cord injury (SCI), traumatic brain injury (TBI) and ischemic stroke (IS)). An electronic research on PubMed database for literature describing cell-, secretome-, and exosome-based therapies was performed on 18 January 2019 using the following keywords: (cell OR cellular OR stem OR cells) AND (transplantation OR transplant OR “Co-Transplantation” OR Cotransplantation OR engraft OR engraftment OR graft OR grafts) AND (therapy OR therapies OR therapeutics OR therapeutic) NOT Review [Publication Type] AND (“spinal cord injury” or “brain injury” or stroke or parkinson) AND (primates OR mice OR mouse OR rat OR rats OR rodent OR “in vivo” OR “clinical Trials” OR humans) for cell-based therapies and (conditioned medium OR conditioned media OR secretory OR “trophic factors” OR Exosomes OR vesicles OR microvesicles OR vesicular OR “Extracellular vesicles” OR “nanovesicles” OR “exosomal”) AND (therapy OR therapies OR therapeutics OR therapeutic) NOT Review[Publication Type] AND (“spinal cord injury” or “brain injury” or stroke or parkinson) AND (primates OR mice OR mouse OR rat OR rats OR rodent OR “in vivo” OR “clinical Trials” OR humans) for secretome- and exosome-based therapies. The search retrieved 3676 articles for cell-based therapies and 682 articles for secretome- and exosome-based therapies. Data extraction was independently performed by two researchers.

This new concept of cell-free-based therapies focused on the cells’ secretome gained momentum with the work of Gnecchi et al. [34]. They demonstrated that the regenerative potential of MSC secretome in a model of heart infarct and inspired secretome application in the most diverse areas of regenerative medicine including the CNS, as seen in Figure 1, with the emergence of publications after 2009. Moreover, as shown in Figure 1, the use of secretome has increased exponentially from 2009 until the present day. More recently, the sole use of exosomes has also emerged as a promising therapeutic approach, as explored in the next sections. This kind of approach overcomes many of the ethical obstacles of cellular transplantation as well as not presenting survival or erroneous differentiation complications of the cells in the host tissue, while preserving the potential of the paracrine action of cellular therapy. 

Given the potential of secretome application to CNS disorders, several advances in this field have recently been made. The following sections discuss some of the major achievements in this new field, with a focus on secretome production, standardization, optimization of the collecting protocols, and its composition.

## 3. Collecting Procedure

Generally, the procedure to obtain secretome from any cellular type follows the same guidelines. Briefly, after a cell culture is established, the growing media (typically supplemented with exogenous factors) is removed and the cells are washed and incubated 24 h to 48 h in a serum-free media with antibiotic (conditioned period). After the conditioned period, only the factors released by the cells (secretome/conditioned media (CM)) are collected and stored at −80°C after being snap-frozen to maintain its biological properties [17,35,36]. In the last decade, new conditioning methodologies have been used to prime the cells to produce different pools of molecules, as detailed below. Methodologies can take advantage of hypoxia, cell activation with apoptotic stimuli, inflammatory stimulus, or even the use of bioreactors. 

Hypoxia. In regular conditions, cellular cultures are maintained at 5% of relative CO_2_ and 21% O_2_. However, under oxygen deprivation, the MSCs alter their secretome properties, enhancing the production of chemoattractant factors (interleukin-8 (IL-8), monocyte chemoattractant protein-1 (MCP-1), RANTES, granulocyte-macrophage colony-stimulating factor (GM-CSF) and angiogenic mediators (angiogenin, matrix metalloproteinase-9 (MMP-9), vascular endothelial growth factor-A (VEGF-A), VEGF-C and elevating inflammatory modulators (IL-1b, IL-6, IL1-15, IL-1Ra) [37,38,39]. This alteration on preconditioned cells is also reported to be related to increases on secretome therapeutic potential. In non-CNS-related pathologies, secretome preconditioned in hypoxia from adipose stem cells (ASCs) is reported to improve recovery in a model of hepatic injury in both in vitro and in vivo, with upper regulation of enzymes with antioxidant properties [40]. In other injury scenarios, this preconditioning method of MSCs seems to potentiate the wound healing and regenerative process provided by the secretome [41,42,43] when delivering the total secretome or only the vesicular fraction [44,45]. In pathologies related to the CNS, using the secretome of cells preconditioned by hypoxia in a rat model of TBI led to a significantly better performance both in motor and cognitive tests together with minor brain damage when compared with normoxic-secretome [46]. 

Inflammatory stimuli. Other methods to modulate the secretome profile of the cells are carried out using inflammatory cytokines; in this case the preconditioning usually results in modulation of the immunoregulatory proteins. Regarding stimulation with tumor necrosis factor alpha (TNF-α), Lee et al. showed an increase of IL-8 and IL-6 when compared with un-preconditioned secretome of ASCs [47]. Actually, the same findings were corroborated later by Heo et al., who also observed beneficial effects on wound healing, angiogenesis, and immune cell infiltration due to those cytokines [48]. Furthermore, TNF-α seems to be a potent inducer of VEGF production in MSCs [49,50]. Even though the mechanism of secretome modulation remains unknown, more works have been performed in this field. It is also known that lipopolysaccharide preconditioning of ASCs increases the levels of the immunomodulators TNF-α and IL-6 as well as increasing the regenerative profile [51] of secretome.

Molecular stimulation. Preconditioning the cells with other molecular stimuli also seems to modulate the secretome composition. As in the case of hydrogen peroxide (H_2_O_2_), when it is provided to ASCs in culture, their secretome shows a better capability to promote cytoprotective and pro-recovery effects on fibroblasts under oxidative stress when compared to ASCs in regular culture [52]. In a case of skin flap, only the exosome fraction was used and it was observed that pre-stimulation with H_2_O_2_ promoted survival and angiogenesis while reducing inflammation when compared with normal ASC secretome [53]. Due to these secretory properties, ASCs stimulated with H_2_O_2_ are currently being employed in a clinical trial with patients with spinal cord injury (NCT02917291).

Additional molecular methods have been used to modulate the secretome profile such as the pre-activation of cells with signals to stimulate the sonic hedgehog (SHH) pathway; in this case, Wharton’s jelly (WJ)-derived mesenchymal stem cell secretome improved their angiogenic potential when compared with control [54]. The use of drugs, as in the case of trimetazidine and diazoxide, also seems to play a role in human embryonic-derived mesenchymal stem cell secretome when preconditioned with a drug; this primed secretome promotes the increase of immunomodulatory molecules by peripheral blood mononuclear cells (PBMCs) such as IL-10, TNF-α, and IL-1β [55]. Improvements in the secretome of ASCs was also observed increasing the secretion of pro-angiogenic, neuroprotective, and anti-inflammatory molecules and increasing the antioxidant capacity of their secretome when preconditioned with deferoxamine [56]. Another innovative approach is to use cellular extracts including cues of the traumatic tissue and to stimulate the cells with the extracts prior to use in vivo on the correspondent disease. In a case of TBI, the traumatic injury-preconditioned secretome increased the differentiation, migration, and maturation of new-born cells, improving the cognitive function after the trauma [57]. In summary, the previous approaches to preconditioning the secretome indicate the immeasurable alternatives available to obtain personalized secretome according to each objective and to target the most varied aspects of CNS pathologies.

Bioreactors. MSC secretome is currently a promising source to treat CNS pathologies in models of injury and disease, as briefly mentioned before and explored in the following sections. Therefore, one challenge of secretome collection is the step from small-scale production to a bigger scale. While the conventional methods (e.g., culture flasks) work perfectly for small applications, this is not feasible for clinical applications. Currently, the secretome of MSCs is obtained from adherent flask cultures, which is very labor intensive. Furthermore, due to the high number of flasks needed, the risk of variability between flasks, as well as possible contaminations during the process, increases significantly [58]. Therefore, different authors have suggested the use of bioreactors to produce secretome. These dynamic culturing systems allow the biological processes to occur under controlled conditions: temperature, pH, medium flow rate, oxygen supply, and control of nutrient and metabolite exchanges [58,59]. Moreover, they allow the expansion to a high number of cells in one vessel with consequent higher secretome production compared with traditional systems [60]. Despite these positive aspects, the bioreactor systems provide a different environment and external stimuli to the cells compared to the adherent flask culture, producing differences in the secretome profile as already reported, which as a last resort could be suitable. Teixeira et al. demonstrated that human bone marrow (hBM)-MSCs expanded in bioreactors improve differentiation of human neural progenitor cells (hNPCs) to neurons when compared with secretome from static cultures in vitro [61]. Additionally, the use of bioreactors improves the neuroregulatory properties of the secretome of those cells, inducing a higher number of NPCs to differentiate into neurons, increasing the number of dopaminergic neurons and improving the motor performance of PD rat models [17]. 

The basic insights provided in this section suggest that cell secretome could be modulated depending on the external cues provided. As such, future research may focus on developing secretome targeted to different needs, enriching secretome in specific biofactors depending on the pathophysiology of the injury and/or disease under study.

In conclusion, despite the therapeutic value associated with secretome in CNS regenerative diseases as described in the following sections, the production methods remain to be standardized and still face some problems when comparing different studies. These problems include the production protocol, use of different concentrations, and differences in the secretome composition. As such, these problems introduce some concern when comparing results between distinct publications. In order to fill those gaps, methods to characterize the secretome composition are already available as is the case of non-target proteomic analysis, which allows researchers to have a wide detection ability and potentially detect any protein present in the secretome [62]. This step is crucial to standardizing techniques and simplifying knowledge to make advances in the secretome field.

## 4. Secretome Composition

MSCs can be obtained from different sources including bone marrow (BM-MSCs), adipose tissue (ASCs), and umbilical cord tissue (Wharton’s jelly mesenchymal stem cells (WJ-MSCs) and human umbilical cord perivascular cells (HUCPVCs), just to highlight the most commonly cited. 

**Figure 2:**
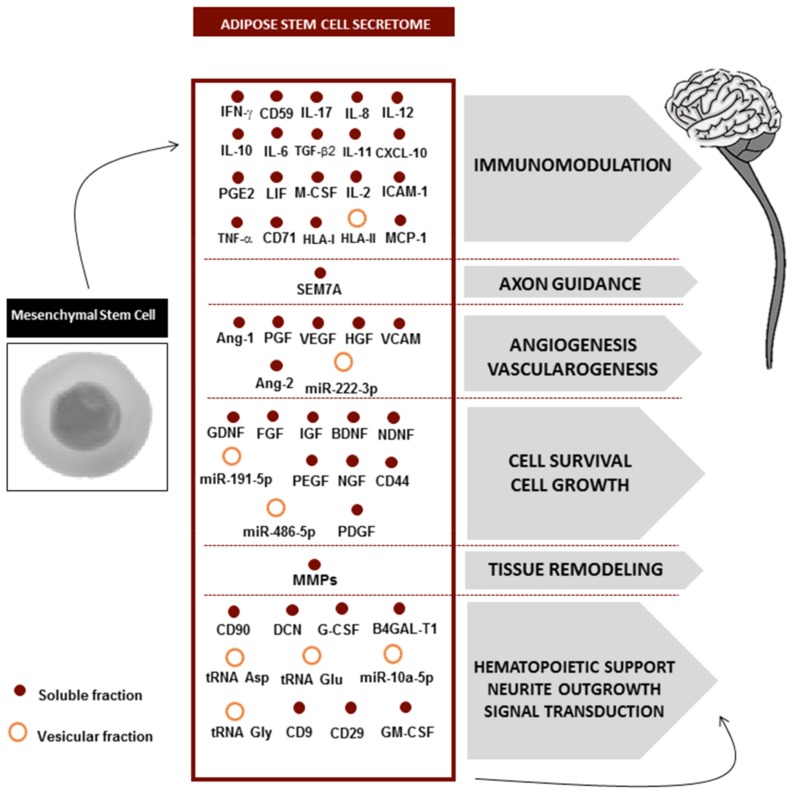
Mesenchymal stem cell secretome composition and their main effects on the CNS. On their soluble fraction (red circles), MSC secretome contains cytokines, chemokines, growth factors, and glycoproteins. On their vesicular fractions (orange circles), the exosomes and microvesicles contain essentially microRNAs. Both the soluble and the vesicular fractions may act on distinct damaged cells of the nervous system, promoting their survival, angiogenesis, neurite outgrowth, and immunomodulation. This information is based on the articles [85,86].

Similarly to what has been reported for BM-MSCs, growth factors such as vascular endothelial growth factor (VEGF), glial-derived neurotrophic factor (GDNF), brain-derived neurotrophic factor (BDNF), hepatocyte growth factor (HGF), fibroblast growth factor (FGF), nerve growth factor (NGF), insulin-like growth factors 1 and 2 (IGF-1 and IGF-2), transforming growth factor beta 1 (TGF- β1), and others have also been found in ASC secretome [62,63] being able to increase neuronal and glial viability [64] (Figure 2). In more detail, VEGF is a mitogen-activated protein that participates in the formation of new blood vessels and in cellular processes, such as proliferation, growth, and survival. Importantly, on the nervous system, VEGF could be associated with neurogenesis [65,66], neuromaturation [67], neuroprotection [68,69], and even neuroregeneration [70]. This effect occurs through VEGF receptor and MAPK/Erk1/2 signal transduction pathways which are mediating the growth effects on CNS neurons [68]. As such, VEGF fosters the reorganization of the spinal motor network after SCI [71] and also underlies a protective effect against ischemia [69]. Among currently known neurotrophic factors, GDNF has the most pronounced neuroprotective effect, which is particularly relevant in ischemic brain damage [72,73] and neurodegenerative diseases [74,75,76]. The effects of GDNF on GluR2-containing α-amino-3-hydroxy-5-methyl-4-isoxazolepropionic acid (AMPA) receptors reduce the postsynaptic membrane permeability to calcium ions, which may be considered a possible mechanism for protecting neural cells from hypoxic damage [73]. This mechanism of GDNF in the maintenance of the main parameters of Ca2+ activity also has a protective effect on the neuron–glia networks and their functional structure [77]. Additionally, by binding to their main receptor (GRFα), GDNF activates several intracellular signaling cascades such as Ras/MAPK and PI3K/Akt, which can suppress pro-apoptotic proteins (p53) and caspases (caspase 3 and 9), promoting neural survival [78]. FGF is a multi-functional growth factor which includes a potent angiogenic potential and plays important roles in the differentiation and function of CNS cells. Importantly, FGF is crucial during CNS development and therefore it may also play an essential role in neurodegenerative disorders [79]. Indeed, the possible manipulation of FGF2/FGFR1 offers therapeutic opportunities for Alzheimer’s [80] and Parkinson’s diseases [81]. 

Moreover, Salgado and coworkers [82] pointed out that the secretome from WJ-MSCs and HUCPVCs is also able to increase survival, proliferation, and differentiation of hippocampal neurons due to the presence of, for example, NGF and FGF [82,83]. Additionally, the BDNF present on these cell secretomes is mediating axonal growth [84]. Figure 2 highlights ASC secretome composition, referring to specific biofactors and their biologic effect on the nervous system.

The cell secretome comprises a myriad of biocomponents and can be divided into two distinct fractions—the soluble fraction (essentially proteins and soluble factors such as cytokines) and the vesicular fraction. Indeed, it is known that the secretome from the different MSC sources contains a soluble fraction rich in pro- and anti-inflammatory cytokines, extracellular matrix proteins, neuroprotective and neurodifferentiation agents, or even neurite growth factors [37,87], as briefly mentioned above. On the other hand, they also present a vesicular fraction, composed of exosomes, microvesicles, and apoptotic bodies [88]. These vesicles containing nucleic acids (e.g., messenger RNAs and microRNAs) and small proteins are secreted through membranal fusion to the extracellular space [89,90]. With this mechanism they are able to modulate the activity of target cells by interacting directly with the cell membrane through an antibody receptor or releasing their biologic content on cell cytoplasm, finally inducing angiogenesis [91,92] and neurite outgrowth [93,94] and regulating inflammatory responses [95,96]. From 2013 onward, research on the use of exosome (nano-scaled vesicles with sizes ranging from 30 to 120 nm)-based therapies has been increasing, as shown also in Figure 1. Exosomes are drawing the attention of the regenerative medicine field because of their therapeutic potential for CNS regeneration [97,98]. Nevertheless, this field is still in its infancy, and as such, more extensive studies are still needed to fully understand the therapeutic potential of these vesicular structures.

Significant knowledge regarding secretome and its constituents has been accumulated over the past few years. However, there are still questions that need to be addressed. It is important to validate and further dissect the individual contribution of either the protein or vesicular fractions of the secretome as compared to the effects that were observed using the total secretome. For instance, are the traditional cytokines and growth factors the main mediators of the paracrine actions promoted by stem cell secretome? Or, instead, are the extracellular vesicles sufficient by acting as transporters of the therapeutic cargo? The answers to these and other questions are now being explored since they are important to the further design of CNS regenerative strategies.

## 5. Secretome Fractions: Where Is the Therapeutic Potential?

Although the cell secretome is now being considered for regenerative medicine, one frequent and important aspect considered about this therapy is its mechanism of action. In this sense, different fractions of the secretome are being thoroughly characterized and analyzed by its immunomodulation and regenerative and/or protective potential. Another important aspect is the possible interaction between different molecules present in the secretome that may act synergistically to potentiate the therapeutic effect. An interesting way of studying the contribution of different fractions to the therapeutic value of the secretome is to separate the soluble factors from the EVs. 

As mentioned previously, several authors have already reported the presence of many proteins in the secretome that have a positive effect on neuronal survival, differentiation, neurite outgrowth, and immunomodulation. Indeed, factors such as GDNF, NGF, HGF, VEGF, IGF-1 and 2, chemokine (C-X-C motif) ligand (CXCL 16), IL-6 and 10, TGF β1, and MCP-1 have been related to the neuro- and immunoregulatory character of MSC secretome [63,87,99,100]. Thus, when applied to CNS disorders, these soluble factors could improve the therapeutic outcomes, as is explored in the next section. This revealed the potential of a single or a group of cytokines and growth factors, acting in coordinated mode to potentiate tissue repair. 

However, the vesicular fraction by itself could also constitute a powerful therapeutic approach since EVs are similarly involved in intercellular communication and may contain all the signals required for a successful contact [101]. Increasing evidence shows that cells modify the EV content in response to the microenvironment, revealing that EVs are involved not only in intracellular signaling but also in the global mechanism of maintaining physiological homeostasis [90]. Therefore, EVs carry nucleic acid and proteins in their lumen or in their lipid membrane to mediate intercellular communication [102,103]. Thus, proteins could also act by the fusion of EVs with recipient cells, allowing the target delivery of soluble factors while maintaining their stability and biological potency as it keeps them protected from microenvironment proteases. Moreover, nucleic acids such as mRNAs, microRNAs, and tRNAs present in EVs may potentially target factors involved in stem cell self-renewal and immune-related mechanisms [104]. The small size of EVs compared to whole cells also offers therapeutic benefits, including easier injection, reduced macrophage phagocytosis, and improved extravasation through the injured site [105]. Some studies have also shown that EVs offer innate biocompatibility, high physical-chemical stability, cell selective fusion, and long-distance communication [106,107,108]. The cargo transference between different cells allows a rapid alteration in gene expression and control of critical processes and cell functions [109]. Additionally, EVs could be chemically and biologically modified to broaden, alter, and/or enhance their therapeutic capability [103,105,110], the engineering of EVs becoming an emerging focus of research. The therapeutic potential of EVs has received the attention of researchers in the field of CNS pathologies such as SCI [53,84,111,112], IS [113,114,115], and PD [92,116,117,118]. Indeed, the administration of MSC-derived exosomes could restore tissue function in multiple disease/injury models [97]. 

Besides the facts mentioned above, the mediators of the secretome therapeutic potential still need to be further clarified. In that context, the impact of each part—soluble and vesicular—should be evaluated in order to understand their individual value, as well as to understand the secretome therapeutic potential for each disorder context, specifying their action to specific dysfunctional mechanisms present on the pathology. The next section explores the therapeutic opportunities offered by the MSC secretome to several neuro disorders, in the context of trauma or neurodegeneration. In each case, the interaction between molecules and vesicles or solely the MSCs’ trophic factors/vesicles could be presented as the mediators of the secretome’s mechanism of action. 

## 6. Secretome Applications to CNS Disorders

The cell secretome is an essential component of both the paracrine and autocrine cell signaling mechanism, having therefore an important role in the regulation of many physiological processes. Additionally, several molecules are present in its constitution that help in neuronal tissue repair either by acting directly on neurons to protect them from further damage and promoting regeneration or by indirectly by mechanisms such as immunomodulation. Its effects on neuronal survival, regeneration, and/or immunomodulation through the action of soluble and vesicular factors are crucial for reducing or even stopping disease progression and for promoting repair [33]. Consequently, there is increasing interest in their study to help understand different pathologies and identify potential therapeutic targets. 

In this section, we analyze the current applications of stem cell secretome as a new tool for the treatment of CNS diseases, focusing for this purpose on MSC secretome, as it is currently the primary source for these applications. Thus, the secretome from the different MSC sources has shown to be a valid tool for the neuroprotection and survival of neuronal and glial cells through the release of a range of multiple trophic factors and vesicles. 

### 6.1. Spinal Cord Injury (SCI)

SCI leads to long-term functional deficits due to the loss of neurons and glial cells, inflammation, and demyelination [18]. The occurrence of the lesion creates a non-permissive inflammatory and chemical environment for neuronal regeneration. The release of immune modulatory and neurotrophic paracrine factors into the lesion site by MSCs could modulate that environment to promote glial scar reduction, immunomodulation, axonal regeneration, and neurite outgrowth. Indeed, the effect of cell transplantation has been proven to be due to the trophic factors as BDNF, HGF, and NGF [119,120,121,122] which were able to promote axonal growth and vascularization after SCI. At the in vitro level, MSC-CM also plays a primary role in skewing pro-inflammatory M1 microglia stimulated with lipopolysaccharide (LPS) toward inflammation-resolving M2 cells [123]. More specifically, secretome from ASCs reduced the production of TNF-α by M1 macrophages while enhancing TGF β1 and IL-10 release by M2 macrophages [124]. in vivo, injecting BM-MSC secretome into the lesion site is reported to promote neuroprotection, leading to reduction of the cystic cavity and preservation of the spinal tracts, leading overall to locomotor recovery of SCI animals [125,126]. Related to the administration solely of the vesicular fraction, MSC exosomes could promote pro-angiogenic and anti-inflammatory effects and axonal regeneration, and suppress glial scar formation and cell apoptosis, attenuating the lesion size and improving functional recovery after traumatic SCI [127,128,129]. Additionally, an intravenous injection of MSCs or MSC-derived exosomes in an SCI context revealed a comparable therapeutic effect [130]. Overall, these findings suggest an interaction between soluble molecules and vesicles secreted by MSCs with neuronal and immune cells, reinforcing the prospective clinical value of using the secretome or the vesicles released by these cells on SCI treatment.

### 6.2. Traumatic Brain Injury (TBI)

TBI is characterized by external mechanical forces causing brain injury, which potentially cause physical, cognitive, and emotional impairments to the patients [131,132]. Due to the immunomodulatory properties of MSCs, their secretome may also be used to modulate the secondary injury mechanisms of TBI, controlling the exaggerated inflammatory response, reducing pro-inflammatory cytokines, and promoting an increase in neural stem cell proliferation and differentiation [133,134,135]. Several factors secreted by MSCs such as NGF, GDNF, BDNF, and Wnt3a could attenuate the loss of cholinergic neurons in the medial septum of mice and still promote hippocampal neurogenesis [136,137]. Furthermore, extracellular vesicles secreted by MSCs have been shown to reduce neuroinflammation and promote neurogenesis and angiogenesis, rescuing spatial learning and motor impairments in TBI animal models [138,139,140,141,142,143]. These effects could be mediated by the miRNA content of those vesicles, which already have been demonstrated to be able to regulate, for example, neurite outgrowth by the transfer of miR-133b to neural cells [90]. These studies may indicate the potential value of MSC secretome as a potential therapy for TBI.

### 6.3. Ischemic Stroke (IS)

IS is a cerebrovascular disease that results from blood vessel occlusion or damage, leading to the suppression of blood supply and therefore resulting in focal tissue loss and death of endothelial and neuronal cells [132]. The application of MSC secretome in this context revealed also that the factors released by these cells such as IGF-α and BDNF could promote neuroprotection, by blocking neuronal damage and tissue loss [144]. Moreover, the MSCs’ paracrine factors inhibit the activation of p38 MAPK and JNK, which attenuate astrocyte injury by GFAP downregulation. Additionally, Chopp’s lab added evidence about the MSC exosomes’ therapeutic potential, demonstrating that the administration of BM-MSC exosomes 24 h after injury resulted in increased neurogenesis and angiogenesis in the ischemic boundary zone [145]. Indeed, the administration of MSC extracellular vesicles led to post ischemic immunosuppression, providing a successful environment for brain remodeling [146,147,148]. 

### 6.4. Parkinson’s Disease (PD)

PD is a neurodegenerative disorder characterized by the progressive degeneration of dopaminergic neurons. As a result, patients develop motor complications such as rigidity, bradykinesia, and postural instability [20]. In PD, cell replacement therapies could be an attractive strategy for replacing the progressive loss of nigral dopaminergic neurons. However, the injection of MSC secretome has already shown to induce beneficial effects, promoting a partial reversion of the PD histological deficits and gains on animals’ motor performance [17]. This could be achieved by the secretion of neurogenic, neurodevelopmental, neurorescuing, or anti-apoptotic factors or, in another point of view, by immunomodulatory and anti-inflammatory mediators present on the MSC secretome. Additionally, Mendes-Pinheiro et al. have shown that, in fact, secretome-based therapies could be more efficient than their cell transplant counterparts, in a similar model of PD [149]. Particularly interesting were the results reported by Hee et al. showing that the secretome from MSCs can reduce the α-synuclein aggregates, one of the hallmarks of the disease, through an MMP-2-based mechanism [150]. 

Based on the cited studies, it is clear that there is increasing evidence that stem cell secretome can have neuroprotective, neuroregenerative, and immunomodulatory actions. Many studies support the conclusion that the recovery of neural and glial function in CNS trauma and disorders is due to the secretion of soluble factors and vesicles by the transplanted MSCs rather than by cell replacement. A study using MSC transplantation in an SCI model also supported this observation as no MSCs were found within the host tissue, but an increase in factors such as NGF could instead be observed [120]. These secreted bioactive products can suppress local inflammation, enhance angiogenesis, inhibit apoptosis, and stimulate proliferation and differentiation of tissue-resident cells. Although most of the described approaches still remain at experimental stages, continuing efforts in developing new secretome-based therapies for CNS disorders will enable the adoption of these techniques into a clinical context.

## 7. Conclusions

Central nervous system dysfunction, caused by either injury or disease, leads to a disruption of the complex network between neuronal and glial cells, having a profound effect on a patient’s life. The restricted regenerative capacity of the adult CNS and the following inhibitory environment make the innate repair process extremely limited. To tackle this limitation, several therapeutic approaches have been tested including pharmacological or cellular therapies. The latter is a promising strategy, although several difficulties related to ethical and safety concerns, tissue availability, and graft rejection concerns must be overcome. As we have reviewed here, a recent paradigm shift has emerged suggesting that these cells might exert beneficial effects through their paracrine effects—the secretome—rather than by cellular replacement. Thus, while early studies primarily attributed the beneficial effects underlying stem cell therapy to its differentiation and replacement competence, it is now known that the therapeutic action is strongly mediated through paracrine factors that include soluble factors and EVs, which (as we pointed out here) apparently provide a supportive environment to host damaged CNS cells and enhance endogenous regenerative processes following injury or disease, promoting significant improvements when compared to vehicle treatments. Furthermore, secretome derivates may represent a considerable advantage over cells when considering manufacturing, storage, and long product shelf life. However, this still presents some obstacles to increasing the robustness of the approach.

Here, we presented the current knowledge regarding production methods used to improve the secretion of certain molecules and the use of bioreactors to scale up the process and standardize the collection methods, to obtain large amounts of secretome. Nevertheless, understanding the mechanism behind the shift in the molecular constitution still remains, and the methods needed to scale up secretome collection must still be widely implemented to decrease the demand on manual processes and increase good manufacturing practices that follow good quality standards. Another interesting aspect to help comprehend and optimize secretome application to CNS disorders is a deeper understanding of the secretome composition, and how each part is interacting with different pathologies. This represents an important step forward and will help understand secretome interaction with different pathologies in a global perspective. As a long shot, the combination of these aspects of the knowledge about the secretome may allow the development of targeted secretome to tackle different pathophysiological deficits in a multidisciplinary way. Nonetheless, secretome as therapy also has limitations concerning especially tissue transport, protein stability, and half-life time. This reinforces the need to develop new delivery strategies. Therefore, some options are currently appearing, such as the coupling of secretome with bioengineered materials to extend the duration of secretome therapeutic effects. Biomaterials are often used since they mimic the native extracellular matrix but also provide structural support to the transplanted factors. A combinatory approach could enhance the positive effects, such as the coupling of biomaterials, cells, and biological factors, which promote accelerated tissue repair. In this review, we showed that the development of secretome-based therapies for CNS disorders is an interesting but still emerging field, with positive results in both smaller mammals and in vitro, dissecting priming methods to modulate the secretome profile, unveiling the secretome composition, and elucidating the different secretome fractions. Those aspects must be further explored so that secretome and its components can be validated as a valuable therapy for CNS disorders.
